# The value of MRI in differentiating ovarian clear cell carcinoma from other adnexal masses with O-RADS MRI scores of 4–5

**DOI:** 10.1186/s13244-024-01860-z

**Published:** 2025-01-29

**Authors:** Lingling Lin, Le Fu, Huawei Wu, Saiming Cheng, Guangquan Chen, Lei Chen, Jun Zhu, Yu Wang, Jiejun Cheng

**Affiliations:** 1 Department of Radiology, Shanghai First Maternity and Infant Hospital, School of Medicine, Tongji University, Shanghai, China; 2 Department of Radiology, Renji Hospital, School of Medicine, Shanghai Jiao Tong University, Shanghai, China; 3 Department of Obstetrics and Gynecology, Shanghai First Maternity and Infant Hospital, School of Medicine, Tongji University, Shanghai, China; 4 Department of Research, Shanghai United Imaging Intelligence Co. Ltd, Shanghai, China; 5 National Engineering Research Center for Nanotechnology, Shanghai, China

**Keywords:** Ovary, Clear cell carcinoma, MRI, Differential diagnosis, Ovarian-adnexal reporting and data system MRI risk stratification system

## Abstract

**Objective:**

To assess the utility of clinical and MRI features in distinguishing ovarian clear cell carcinoma (CCC) from adnexal masses with ovarian-adnexal reporting and data system (O-RADS) MRI scores of 4–5.

**Methods:**

This retrospective study included 850 patients with indeterminate adnexal masses on ultrasound. Two radiologists evaluated all preoperative MRIs using the O-RADS MRI risk stratification system. Patients with O-RADS MRI scores of 4–5 were divided into a training set (*n* = 135, hospital A) and a test set (*n* = 86, hospital B). Clinical and MRI features were compared between CCC and non-CCC patients. Analysis of variance and support vector machine were used to develop four CCC prediction models. Tenfold cross-validation was applied to determine the hyperparameters. Model performance was evaluated by the area under the curve (AUC) and decision curve.

**Results:**

221 patients were included (30 CCCs, 191 non-CCCs). CA125, HE4, CEA, ROMA, endometriosis, shape, parity, unilocular, component, the growth pattern of mural nodules, high signal on T1WI, number of nodules, the ratio of signal intensity, and the ADC value were significantly different between CCCs and non-CCCs. The kappa and interobserver correlation coefficient of each MRI feature exceeded 0.85. The comprehensive model combining clinical and MRI features had a greater AUC than the clinical model and tumour maker model (0.92 vs 0.66 and 0.78 in the test set; both *p* < 0.05), displaying improved net benefit.

**Conclusions:**

The comprehensive model combining clinical and MRI features can effectively differentiate CCC from adnexal masses with O-RADS MRI scores of 4–5.

**Critical relevance statement:**

CCC has a high incidence rate in Asians and has limited sensitivity to platinum chemotherapy. This comprehensive model improves CCC prediction ability and clinical applicability for facilitating individualised clinical decision-making.

**Key Points:**

- Identifying ovarian CCC preoperatively is beneficial for treatment planning.
- Ovarian CCC tends to be high-signal on T1WI, unilocular, big size, with endometriosis and low CEA.
- This model, integrating clinical and MRI features, can differentiate ovarian CCC from adnexal masses with O-RADS MRI scores 4–5.

**Graphical Abstract:**

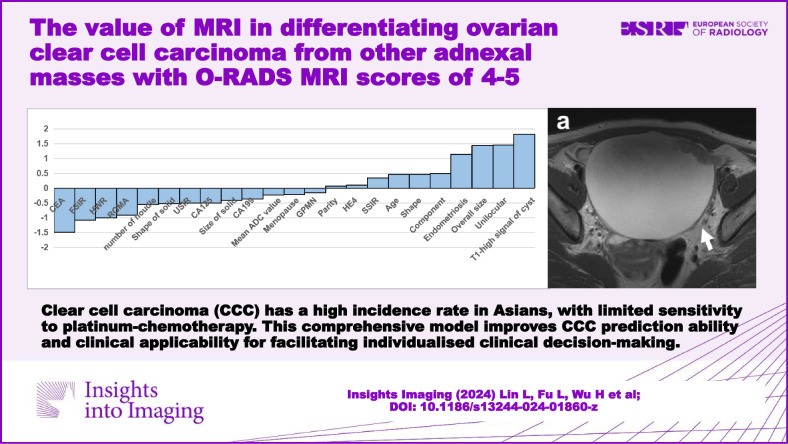

## Introduction

Ovarian cancer (OC) has the highest mortality rate among cancers of the female reproductive system [1]. Epithelial OC is the main type of OC, and clear cell carcinoma (CCC) accounts for 10% [2]. The prevalence of CCC is notably higher in Asia and is associated with increased venous thrombosis [2, 3]. Moreover, the response rate of CCC to platinum-based chemotherapy is notably lower than that of other OC subtypes, with rates ranging from only 11% to 50%, resulting in a poor prognosis [3, 4]. For OC patients who cannot achieve satisfactory tumour reduction, platinum-based chemotherapy for reducing the tumour volume before surgery is recommended [2, 4]. Preoperative identification of CCC, which is insensitive to platinum, can prevent unnecessary use of platinum drugs. In OC patients undergoing initial surgery, preoperative identification of CCC can encourage patients to receive pulmonary CTA, which helps reduce the surgical risks associated with pulmonary embolism. It can also assist surgeons in formulating a comprehensive surgical strategy and facilitate timely personalised treatment. Thus, accurately distinguishing CCC preoperatively is crucial for clinical decision-making.

Imaging is the preferred non-invasive and cost-effective approach for the preoperative differential diagnosis of CCC, with several studies investigating its value [5–9]. A study compared preoperative MRI features of CCC and high-grade serous carcinoma, indicating that CCC typically presents as an oval, unilocular cystic mass with large papillary projections and T1-hyperintense cystic components [6]. However, the study did not evaluate which MRI feature is most critical for distinguishing CCC or rank the features by their importance in differentiation. A model combining clinical and imaging characteristics outperforms those using a single variable in predicting CCC, but its external validation and clinical benefits remain unexplored [8]. A radiomic CCC prediction model based on CT data was developed with an area under the curve (AUC) of 0.86, but the radiomic features have poor interpretability [9]. Currently, research on the preoperative imaging of CCC has several limitations, such as the scattered exploration of image features, unclear proportions of features, and poor interpretability. This study summarises the corresponding CCC imaging features from several previous representative studies to construct CCC prediction models based on samples from two hospitals [5–8]. It is also necessary to confirm the proportion of each feature in the model and highlight the features that are important to identify CCC.

As a highly malignant tumour, almost all CCCs have enhanced solid components within the mass on routine pelvic enhanced MRI, which can be scored as 4–5 points in the ovarian-adnexal reporting and data system (O-RADS) MRI risk stratification system. Considering MRI can display the internal components and adjacent structure of the adnexal mass, Thomassin-Naggara et al [10] proposed the O-RADS MRI risk stratification system to preoperatively distinguish between benign and malignant adnexal masses. Adnexal masses with O-RADS MRI scores of 4–5 have a positive predictive value (PPV) of 90% for malignancy. Therefore, differentiating CCC from adnexal masses with O-RADS MRI scores of 4–5 narrows the scope of predicting CCC, and avoids selection bias. In this study, we incorporate the O-RADS MRI risk stratification system as part of the CCC preoperative evaluation strategy, which will not only help radiologists conduct prospective evaluations but also standardise the differentiation process and promote interdisciplinary exchanges. However, based on the O-RADS MRI risk stratification system, improvements in the accuracy (AC) of distinguishing CCC from adnexal masses have not yet been discussed.

Thus, we aimed to construct a model by combining clinical characteristics with MRI features for differentiating CCC from adnexal masses with O-RADS MRI scores of 4–5.

## Methods

### Patients

**Fig. 1:**
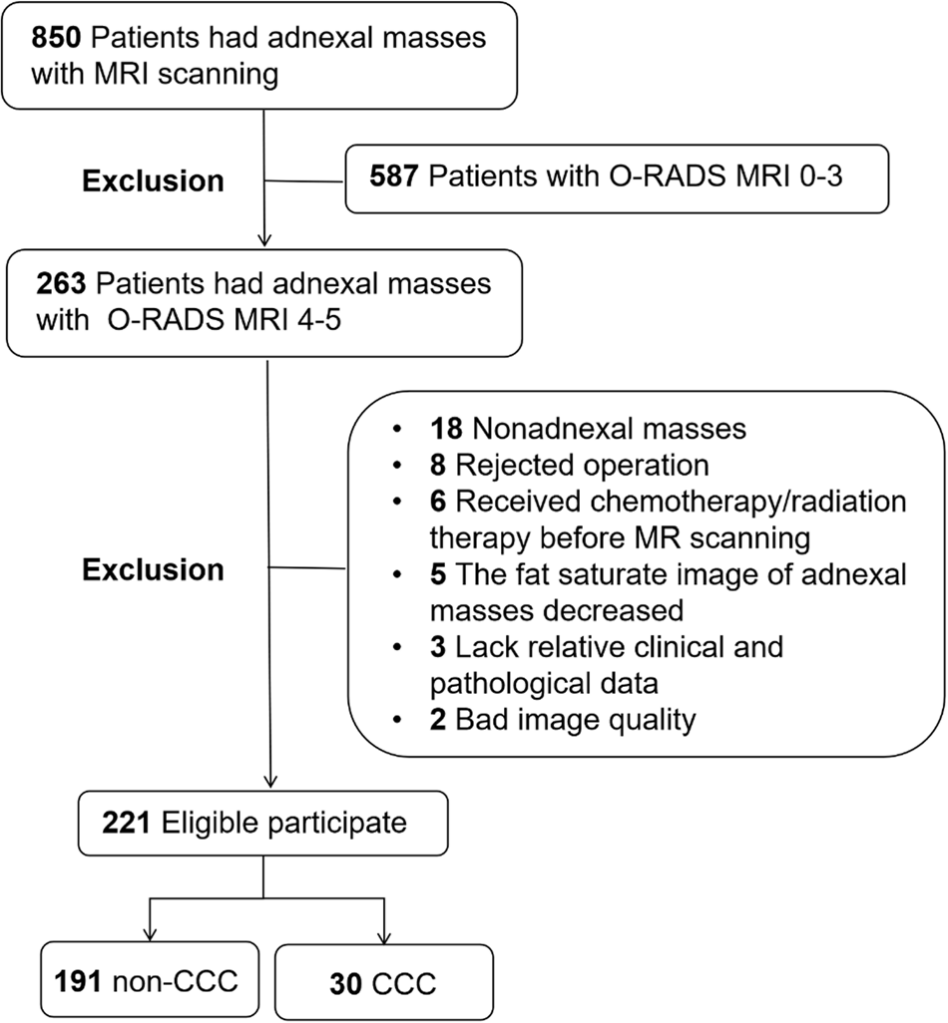
Flowchart of the study population

The study was performed after approval from the institution’s medical research committee. We initially identified 850 women with indeterminate adnexal masses via ultrasound examination and then performed MR scans at Hospital A (Renji Hospital, School of Medicine, Shanghai Jiao Tong University; between January 2020 and March 2022) and Hospital B (Shanghai First Maternity and Infant Hospital, School of Medicine, Tongji University; between January 2020 and February 2021). The exclusion criteria were as follows: 1. the O-RADS MRI score ranged from 1 to 3 (*n* = 587); 2. rejected operation (*n* = 8); 3. non-adnexal masses (*n* = 18); 4. decreased fat saturation of the adnexal masses (*n* = 5); 5. received chemotherapy or radiation therapy before MR scanning (*n* = 6); 6. lack of relative clinical and pathological data (*n* = 3); and 7. bad image quality (*n* = 2). Overall, 222 patients with O-RADS MRI scores of 4–5 were divided into a training set (*n* = 135, hospital A) and a test set (*n* = 86, hospital B). The flowchart of the study population is shown in Fig. 1. Clinical data included age, menopausal status, and a history of endometriosis. Serum tumour markers, such as CA125, HE4, CEA, CA199, and ROMA, were obtained within one week before surgery.

### MR imaging protocol

MR scanning was performed on a 1.5-Tesla system (GE Medical Systems) at Hospital B and a 3.0-Tesla system (GE and Siemens Medical Systems) at Hospital A. The MR imaging protocol mainly included sagittal T2-weighted imaging (T2WI), axial T2WI, fat-suppressed T2WI (FS-T2WI), axial T1-weighted imaging (T1WI), and gadolinium-enhanced TIWI. Patients were administered an intravenous injection of gadopentetic acid (0.2 mmol/kg). The apparent diffusion coefficient (ADC) was derived from two values of b (0 s/mm^2^ and 800 s/mm^2^). The details are listed in Tables S1–2.

### MR imaging analysis

Two board-certified radiologists with eighteen and eight years of experience in pelvic MRI independently read and recorded each image. The assessment provided by the senior radiologist was considered the standard in cases of disagreement between the two observers. The radiologists were unaware of the patient’s pathological results but used the O-RADS MR score developed by Thomassin et al [10, 11] for each adnexal mass evaluation. The criteria for MRI features were based on several previously published terms related to CCC (Table 1). The overall condition of the tumour was evaluated by shape, parity, unilocularity, component, and overall size. The internal details of the tumour were evaluated by the shape of the solid, size of the solid portion, number of nodules, growth pattern of mural nodules (GPMN), high signal on T1WI, the ratio of height to width of solid components (HWR), the signal intensity ratio of solid (SSIR), signal intensity ratio of fluid (FSIR), and ADC value [12]. Enhanced assessment refers to the signal intensity ratio of outer myometrium to tumour solid tissue in the arterial phase (USIR).

**Table 1:** Definition of MRI qualitative and quantitative features

Term	Reference	Definition
MRI qualitative features		
Shape	Ma et al [6]	“Regular” meant lesions tended to be oval with smooth boundaries; others were divided into “irregular”.
Parity	Morioka et al [7]	“Nultipara” meant lesion from one ovary or fallopian tube; “Mullipara” meant lesion from two ovaries or fallopian tubes.
Unilocular	Ma et al [6]	“Present” meant cystic lesion without septa; “Absent” meant cystic lesion with septa.
Component	Caroline et al [10]	“Cyst” meant lesion consists of < 20% solid tissue; “Solid” meant lesion consists of at least 80% solid tissue; “Mixed” meant lesion consists of 20–80% solid tissue.
Shape of solid	Ma et al [6], Morioka et al [7], Li et al [8]	“Nodule” meant the solid tissue only consisted of nodules, with a long diameter < 2 cm; “Mass” meant the solid tissue only consisted of nodules, with a long diameter > 2cm; “mixed” meant the solid tissue consisted of nodules and mass.
GPMN	Morioka et al [7], Li et al [8]	“Eccentric” meant broad-based nodular structures; “Concentric” meant polypoid structures and polypoid structures fused into clusters.
High signal on T1WI	Ma et al [6]	Meant T1-hyperintense in the cystic component.
MRI quantitative feature		
Overall size of tumour	Yasuhisa et al [5]	Meant the longest diameter of the general tumour.
Solid portion size	Yasuhisa et al [5]	Meant the longest diameter of the solid portion.
Number of nodules	Morioka et al [7]	Meant the number of nodules in the tumour.
HWR	Morioka et al [7], Li et al [8]	Meant the ratio of height to width of solid components.
SSIR	Yasuhisa et al [5]	Meant the signal intensity ratio of the tumour solid component to the bladder in T2WI.
FSIR	Yasuhisa et al [5]	Meant the signal intensity ratio of the tumour fluid component to the iliopsoas muscle in the same layer in T1WI.
USIR	Yasuhisa et al [5]	Meant the signal intensity ratio of outer myometrium to tumour solid tissue in the arterial phase.
ADC value	Yasuhisa et al [5]; El et al [12]	The minimum ADC value was the lowest value among the five values; The mean ADC value was the average of the five values.

### Statistical analysis

For continuous variables with a normal distribution, the means and standard deviations were provided, whereas medians (ranges) were reported for nonnormally distributed data. For continuous variables, the independent samples *t*-test was used to compare differences with a normal distribution, whereas the Mann‒Whitney *U*-test was used for nonnormally distributed data. Pearson’s chi-squared tests were used to test the associations between categorical variables.

Up-sampling was carried out by repeating random cases to balance the positive and negative samples to fix the imbalance of the training dataset. If the Pearson correlation coefficient (PCC) of a feature pair exceeded 0.80, one of the features was removed to reduce multicollinearity and prevent model overfitting [13]. Before building the model, analysis of variance was used to select features. The support vector machine (SVM) was used as the classifier to build the model. Tenfold cross-validation was applied to determine the hyperparameters of the model in the training cohort. The AUC of the test set was used to determine the number of model features. The model’s performance was evaluated by the AUC, decision curve analysis (DCA), and calibration curve. The AC, sensitivity (SE), specificity (SP), PPV, and negative predictive value (NPV) were also calculated at a cut-off value that maximised the value of the Yorden index. The 95% confidence intervals (CIs) were estimated by bootstrapping with 1000 samples. The DeLong test was used to compare the AUCs.

Interobserver agreement regarding the imaging analyses was assessed using Cohen’s kappa coefficient and interobserver correlation coefficient (ICC). Interobserver agreement was defined as slight agreement (0.00–0.40), moderate agreement (0.41–0.60), substantial agreement (0.61–0.80), or almost perfect agreement (0.81–1.00) [14]. All the above processes were implemented with R software (version 4.0.4, http://www.R-project.org) and FeAtureExplorer Pro (FAE, V 0.5.5) on Python (3.7.6) [15]. A *p*-value < 0.05 was considered statistically significant.

## Results

### Clinical and pathological characteristics

The clinical and pathological features of CCC patients and non-CCC patients are compared and analysed in Table 2. The median (range) age was 56 (68) in non-CCC patients and 53.5 (44) in CCC patients (*p* = 0.40). The non-CCC cohort included malignant tumours, borderline tumours, and benign tumours, accounting for 84.82%, 4.19%, and 10.99%, respectively. A total of 162 patients were diagnosed with malignant tumours, which included high-grade serous carcinoma, endometrioid carcinoma, mucinous adenocarcinoma, and other types. Additionally, there were eight patients with borderline tumours and 21 patients with benign tumours, which comprised sex cord-stromal tumours, germ cell tumours, tuberculosis, and mesosalpinx fibroids. There were significant differences in endometriosis, CA125, HE4, CEA, ROMA, ascites, peritoneal plant, Ki-67, and FIGO stage (all *p* < 0.05). The probability of having a history of endometriosis in the CCC group was higher than that in the non-CCC group. Compared with non-CCC patients, CCC patients had lower CA125, HE4, CEA, and ROMA levels. The frequencies of ascites and peritoneal plants were lower in CCC patients (23.3% and 13.3%, respectively). Patients with CCC also had lower Ki-67 levels. Most of the patients with CCC were diagnosed at early stages (I, II), accounting for 80% of the patients. Most non-CCC patients were diagnosed at advanced stages (III, IV), accounting for 56.5%. Postmenopause, CA199, R0 resection, coexisting endometrial carcinoma, P53 mutation, and O-RADS MRI score were not significantly different between the two groups.

**Table 2:** Comparison of clinical and pathological features between CCC and non-CCC

	Non-CCC (*n* = 191)	CCC (*n* = 30)	*p*-value
Age	56 (68)	53.5 (44)	0.40
Postmenopause	105 (55)	13 (43.3)	0.32
Endometriosis	11 (5.8)	12 (40)	< 0.001
CA125	281 (36,238.82)	48.15 (10,193.76)	< 0.001
HE4	156 (6048.17)	55.35 (5.19)	< 0.001
CEA	1.5 (318.70)	1.31 (5.19)	0.04
CA199	12.10 (9998.81)	15.01 (883.66)	0.40
ROMA	0.8 (1.00)	0.16 (0.97)	< 0.001
R0 resection	163 (85.3)	28 (93.3)	0.37
Pathological type			NA
Malignant	162 (84.8)	30 (100)	
High-grade serous carcinoma	114 (59.7)	NA	
Endometrioid carcinoma	28 (14.7)	NA	
Mucinous adenocarcinoma	9 (4.7)	NA	
Other	11 (5.8)	NA	
Borderline	8 (4.2)	NA	
Benign	21 (11)	NA	
Sex cord-stromal tumour	12 (6.3)	NA	
Germ cell tumour	7 (3.7)	NA	
Tuberculosis and mesosalpinx fibroid	2 (1.0)	NA	
Ascites	90 (47.1)	7 (23.3)	0.04
Peritoneal plant	101 (52.9)	4 (13.3)	< 0.001
Endometrial carcinoma	16 (8.4)	4 (13.3)	0.6
Ki-67	0.6 (0.9)	0.4 (0.8)	0.01
P53 mutation	102 (71.3)	22 (75.9)	0.79
FIGO stage			< 0.001
Early	52 (27.2)	24 (80)	
Advanced	108 (56.5)	6 (20)	
O-RADS MRI score			0.052
4	104 (54.5)	22 (73.3)	
5	87 (45.5)	8 (26.7)	

Continuous variables showed as medians (ranges), categorical variables showed as frequency (percentage)

The non-CCC group lacked Ki-67 data from 45 patients and P53 data from 47 patients; CCC group lacked Ki-67 data from 1 patient and P53 data from 1 patient; and O-RADS MRI score: the ovarian-adnexal reporting and data system MRI score

*ROMA* risk of ovarian malignancy algorithm, *R0 resection* no residual lesions after surgery, *Endometrial carcinoma* combination with endometrial carcinoma, *Early stages* FIGO stage I–II, *Advance stages* FIGO stage III–IV, 29 patients with borderline/benign tumour were excluded

### MRI qualitative feature analysis

The results of the qualitative MRI measurements of the CCC and non-CCC patients are summarised in Table 3. There were significant differences in shape, parity, unilocular, component, shape of solid, GPMN, and high signal on T1WI between non-CCC and CCC (all *p* < 0.05). In general view, most CCCs had unilateral lesions (27/30, 90%), regular shape (20/30, 66.7%), unilocular tumour (21/30, 70%) and mixed component (16/30, 53.3%). While most non-CCCs had unilateral lesions (111/191, 58.1%), irregular shape (119/191, 62.3%), unilocular tumour (147/191, 77%) and solid components (80/191, 41.9%). From a detailed perspective, most CCCs had a nodule shape solid (25/30, 83.3%), eccentric nodules (20/30, 66.7%), and high signal on T1WI (22/30, 73.3%). While most non-CCCs had nodule shapes of solid (118/191, 61.8%), concentric nodules (132/191, 69.1%), and lack of high signal on T1WI (149/191, 78%).

**Table 3:** Comparison of qualitative MR features between CCC and non-CCC

	Non-CCC (*n* = 191)	CCC (*n* = 30)	*p*-value
Shape			< 0.001
Regular	72 (37.7)	20 (66.7)	
Irregular	119 (62.3)	10 (33.3)	
Parity			< 0.001
Nultipara	111 (58.1)	27 (90)	
Mullipara	80 (41.9)	3 (10)	
Unilocular			< 0.001
Absent	147 (77)	9 (30)	
Present	44 (23)	21 (70)	
Component			0.02
Cyst	33 (17.3)	9 (30)	
Solid	80 (41.9)	5 (16.7)	
Mixed	78 (40.8)	16 (53.3)	
Shape of solid			0.05
Nodule	118 (61.8)	25 (83.3)	
Mass	37 (19.4)	1 (3.3)	
Mixed	36 (18.8)	4 (13.3)	
GPMN			< 0.001
Eccentric	59 (30.9)	20 (66.7)	
Concentric	132 (69.1)	10 (33.3)	
High signal on T1WI			< 0.001
Absent	149 (78)	8 (26.7)	
Present	42 (22)	22 (73.3)	

Categorical variables showed as frequency (percentage)

### MRI quantitative feature analysis

The results of the quantitative MRI measurements of the CCC and non-CCC patients are summarised in Table 4. The minimum and mean ADC values were significantly higher in CCC patients (1.04 (0.92) and 1.04 (0.92)) than in the non-CCC patients (0.85 (1.06) and 0.9 (1.04)) (both *p* < 0.05). There were fewer nodules in the CCC group than in the non-CCC group (2 (11) vs 4 (19)) (*p* < 0.05). Compared with non-CCC patients, the CCC patients had a lower USIR (0.77 (1.67) vs 0.98 (1.84)) (*p* < 0.05) and higher FSIR (0.71 (2.08) vs 0.65 (5.38)) (*p* < 0.05). However, the overall size, solid portion size, HWR, and SSIR were not significantly different between CCC and non-CCC patients.

**Table 4:** Comparison of quantitative MR features between CCC and non-CCC

	Non-CCC (*n* = 191)	CCC (*n* = 30)	*p*-value
Minimum ADC value	0.85 (1.06)	1.04 (0.92)	< 0.001
Mean ADC value	0.9 (1.04)	1.04 (0.92)	< 0.001
Overall size (mm)	700 (2900)	949 (2270)	0.07
Solid portion size (mm)	30 (848)	27.17 (79.4)	0.64
Number of nodules	4 (19)	2 (11)	< 0.001
HWR	1.25 (2.24)	1.17 (2)	0.50
SSIR	0.49 (1.00)	0.46 (0.88)	0.58
FSIR	0.65 (5.38)	0.71 (2.08)	0.04
USIR	0.98 (1.84)	0.77 (1.67)	0.03

Continuous variables showed as medians (ranges)

*ADC* apparent diffusion coefficient 10^−3^ mm^2^, *HWR* meant the ratio of height to width of solid components, *SSIR* solid signal intensity ratio, *FSIR* fluid signal intensity ratio, *USIR* uterine signal intensity ratio

**Fig. 2:**
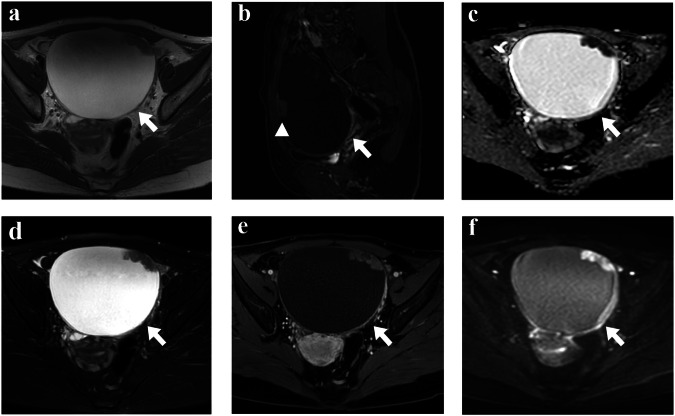
The MR images of a 35-year-old CCC female (FIGO IA stage) with endometriosis were shown as follows. Axial T2WI and axial FS-T2WI (**a**, **d**), demonstrated the left ovary had a big unilocular cystic mass with a eccentric nodule (arrowhead), that had a prominent enhancement nodule and little line-like high signal on CE-T1WI with FS (**b**, **e**). Axial DWI showed the nodule had a high signal (**f**), while it had a low signal at ADC map (**c**)

**Fig. 3:**
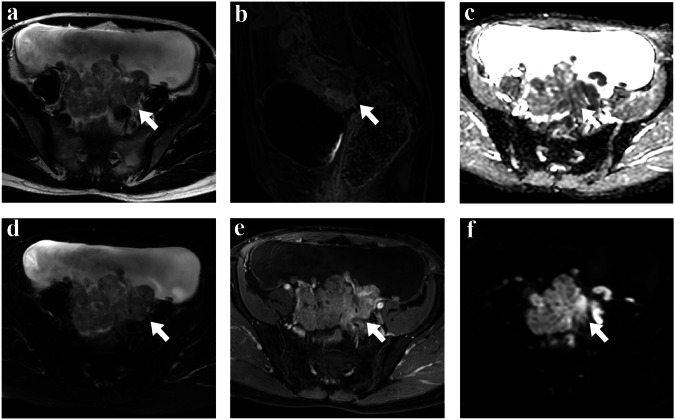
The MR images of a 54-year-old non-CCC (high-grade serous carcinoma) female (FIGO IVB stage) with massive ascites were shown as follows. Axial T2WI and axial FS-T2WI (**a**, **d**), demonstrated the left and right ovary had multiple solid nodules fusing into clusters (arrowhead), that had prominent enhancement on CE-T1WI with FS (**b**, **e**). Axial DWI showed the solid tissues had high signal (**f**), while they had low signal at ADC map (**c**)

The interobserver agreement of each MRI feature was almost perfect (all the kappa and ICC values were above 0.85) (Table S3). Examples of CCCs and non-CCCs are shown in Figs. 2 and 3.

### Comparison of each CCC prediction model with SVM

**Fig. 4:**
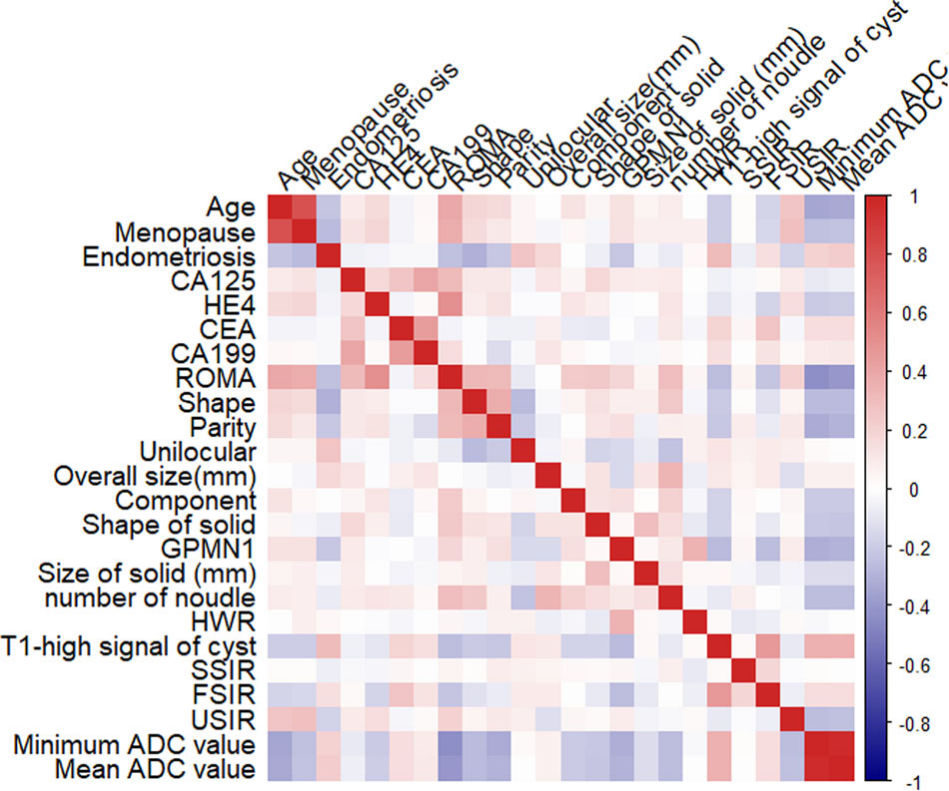
The correlation between each parameter was shown in the heatmap

There were no significant differences in any of the clinical or MRI parameters between the training set and testing set (Table S4). The correlation between each parameter is shown in the heatmap (Fig. 4). The diagnostic performance of the four CCC diagnostic models is shown in Table 5.

**Table 5:** Diagnostic performance of each CCC diagnostic model

Model	Train set AUC (95% CI)	Validation set AUC (95% CI)	Test set AUC (95% CI)	FN	AC	SE	SP	PPV	NPV
Clinical model	0.70 (0.55–0.85)	0.67 (0.64–0.69)	0.66 (0.45–0.86)	3	0.85	0.42	0.92	0.45	0.91
Tumour marker model	0.69 (0.58–0.80)	0.68 (0.66–0.70)	0.78 (0.62–0.94)	5	0.69	0.83	0.66	0.28	0.96
MRI model	0.92 (0.85–0.98)	0.93 (0.92–1.00)	0.87 (0.72–1.00)	15	0.86	0.75	0.88	0.5	0.96
Clinical + tumour marker + MRI model	0.93 (0.84–1.00)	0.93 (0.92–1.00)	0.92 (0.82–1.00)	23	0.92	0.75	0.95	0.69	0.96

*FN* feature number, *AC* accuracy, *SE* sensitivity, *SP* specificity, *PPV* positive predictive value, *NPV* negative predictive value, *95% CI* the 95% confidence interval, *MRI model* conventional MRI + enhanced MRI + ADC model

The clinical model utilised age, postmenopause, and endometriosis, with an AUC of 0.66 (0.45–0.86) in the test set. The tumour marker model comprised CA125, HE4, CEA, CA199, and ROMA, with an AUC of 0.78 (0.62–0.94) in the test set. The MRI model was developed using 13 conventional MRI features (shape, parity, unilocular, component, shape of solid, GPMN, high signal on T1WI, overall size, solid portion size, HWR, number of nodules, SSIR, and FSIR), as well as an enhanced MRI feature (USIR) and a functional MRI feature (ADC value), achieving an AUC of 0.87 (0.72–1.00).

To establish the clinical + tumour marker + MRI model (comprehensive model), we utilised three clinical features (age, postmenopause, and endometriosis), five tumour marker features (CA125, HE4, CEA, CA199, and ROMA), and 15 MRI features (shape, parity, unilocular, component, the shape of solid, GPMN, high signal on T1WI, overall size, solid portion size, HWR, number of nodules, SSIR, FSIR, USIR, mean ADC value). The comprehensive model achieved AUCs of 0.93 (0.84–1.00), 0.93 (0.92–1.00), and 0.92 (0.82–1.00) in the training, validation, and test sets, respectively. In the test set, the AC, SE, SP, PPV, and NPV were 0.92, 0.75, 0.95, 0.69 and 0.96, respectively.

**Fig. 5:**
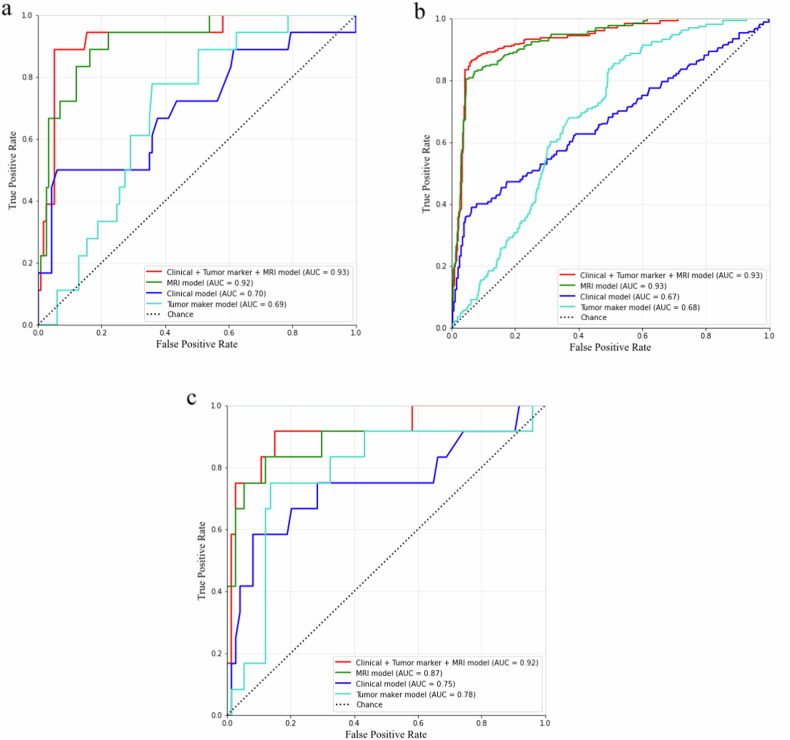
The AUC of four CCC prediction models in the train, validation and test set, respectively (**a**–**c**)

**Fig. 6:**
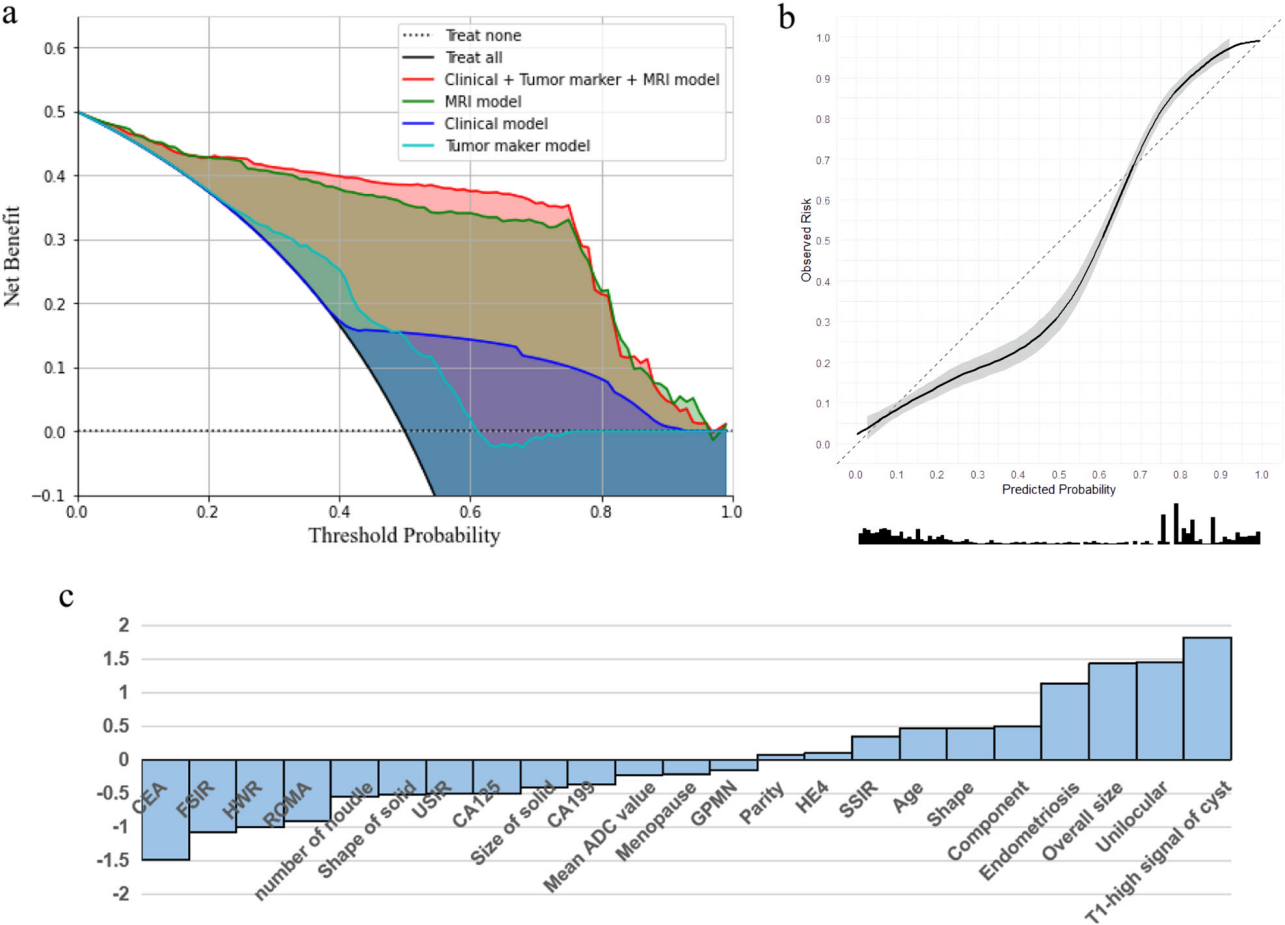
The DCA of four CCC prediction models in the validation set, respectively (**a**). The calibration curves of the comprehensive model in the validation set (**b**). The composition of the comprehensive model (**c**)

The AUCs of each model in the training, validation, and test sets are shown in Fig. 5a–c. The AUCs of the comprehensive model were higher than clinical and tumour marker models in all sets according to De Long’s test (all *p* < 0.05). The DCA and calibration curves of the models are shown in Fig. 6a, b. The comprehensive model had better DCA than the others, and it had good calibration ability in the validation set. The composition of the comprehensive model is listed in Fig. 6c.

## Discussion

In this study, the comprehensive CCC prediction model performed well in the training, validation, and external test sets (the AUCs were 0.93, 0.93, and 0.92, respectively). The NPV of the comprehensive model was 0.96, and the high NPV indicated that the model could reduce the probability of misdiagnosis of CCC before surgery, avoiding delaying platinum-based neoadjuvant chemotherapy. In particular, patients in poor physical condition who are unable to receive surgery, as well as patients whose tumours cannot be completely removed through surgery, can benefit more from this model.

From the feature contribution histogram of the comprehensive model, we can see that the contribution coefficients of seven features exceed one, making a significant contribution to the model. Among these features, the contributions of five features were particularly prominent, namely size, unilocular, high signal on T1WI, endometriosis and CEA, which were similar to the findings of previous studies [6, 7]. Therefore, among adnexal masses with O-RADS MRI scores of 4–5, CCC tends to be a large unilocular cyst with high signal on T1WI, endometriosis and low CEA. In addition, the cases of diagnostic errors in the CCC prediction model were also analysed in the test set. There were four patients who were false positives and erroneously diagnosed as having CCC. Among these patients, three had endometriosis, two had unilocular cysts, and two had high-signal T1W images. This finding prompted us to distinguish between CCC and high-grade serous ovarian carcinoma, and endometrioid ovarian carcinoma when using this model. Additionally, three patients had false-negative results. Among these patients, two lacked high signal on T1WI, two were not unilocular, one displayed elevated CEA and one patient with a lesion which was too small to accurately assess. This finding suggests that the possibility of CCC, even when the tumour is multilocular or lacks a high signal on T1WI, cannot be completely ruled out.

In the face of scattered information, synthesising and building a model could help doctors make more informed and accurate diagnoses [16]. There are several questions on how to select parameters for model development and how to explore and reflect the clinical benefits of the model. According to the previous studies, all the features explored in this study were effective in identifying CCC. Given the significance of each feature, parameters with *p*-values > 0.05 were not excluded. Instead, parameters with a PCC > 0.8 were excluded, prioritising the elimination of features exhibiting high multicollinearity while preserving all potentially valuable features for analysis [13]. The calibration curve and DCA were calculated to evaluate the clinical benefits of the model [17, 18]. The results demonstrated a favourable performance of the comprehensive model.

According to the univariate analysis of qualitative MRI features, CCCs were mostly unilateral regular unilocular cystic tumours with few nodules, similar to previous findings [6, 7]. CCC is related to endometriosis, confirming the theory that it is an endometriosis-related malignant ovarian tumour. Endometriosis may introduce intracystic haemorrhage, which represents a high signal on T1WI and improves intracystic signals [5, 7]. In this study, CCC often exhibited a high signal on T1WI, with a greater FSIR. On the other hand, Morioka et al [7] proposed that CCC cells that arise from HNF-1β-positive premalignant endometriotic cells can form focal and eccentric lesions. Most of the non-CCC cases were malignant tumours, accounting for 85% of the cases. Non-CCC was related to high ki-67, which could explain its high invasiveness. It develops rapidly once it becomes a tumour, possibly resulting in concentric nodules and the fusion of most concentric structures [19, 20]. The same phenomenon could also be observed in CCCs with eccentric nodules, non-CCCs with concentric nodules, and the fusion of concentric structures in this study. Therefore, CCC tends to be diagnosed at an early FIGO stage with fewer ascites and fewer peritoneal plants. However, age, menopausal status, shape, parity, and component contribute relatively less to the CCC differentiation. In terms of tumour markers, we have observed that CEA exhibits a remarkable discriminatory ability in identifying CCC, which aligns with prior research findings [8]. On the other hand, CA125, HE4, and ROMA are not prominent in the differentiation.

According to the univariate analysis of the quantitative MR features, our study revealed that the minimum and mean ADC values were higher for CCC, and the cut-off values were both 1.04 × 10^−3^, indicating reasonable restriction. CCC may have fewer blood vessels than other epithelial OCs due to its indolent behaviour, and less enhancement could theoretically be observed [21]. Previous studies on enhancement were limited to the simple three categories of enhancement, which may miss some useful information [6]. Therefore, the USIR was chosen for the first time to explore the features of CCC. The results showed that CCC had a lower USIR, which was consistent with the biological behaviour of CCC. The number of nodules, HWR, solid portion size, and SSIR were not able to discriminate CCC from the adnexal masses, as observed in other studies [7, 20]. This may have resulted from the inferior reproducibility of the measurement methods or inclusion bias, so more supporting evidence is needed.

The limitations of this study are as follows. The MR images of the training and test sets came from different types of MR scanners. However, the good results of the test set indicate that this CCC prediction model is suitable for different types of MR images. In addition, we scored adnexal masses on routine pelvic MR images using the O-RADS risk system, which may introduce a few benign adnexal masses containing an enhanced solid component divided into O-RADS 4–5, causing interference with the CCC diagnosis. Dynamic contrast-enhanced MR images will be explored in the future.

## Conclusion

In conclusion, this study revealed a valuable model for distinguishing CCCs from adnexal masses with O-RADS MRI scores of 4–5.

## Supplementary information

ELECTRONIC SUPPLEMENTARY MATERIAL

## Data Availability

The experimental data used to support the findings of this study are available from the corresponding author upon request.

## Abbreviations

AC: Accuracy
ADC: Apparent diffusion coefficient
AUC: Area under the curve
CCC: Clear cell carcinoma
CI: Confidence interval
Comprehensive model: Clinical + tumour marker + MRI model
DCA: Decision curve analysis
FS-T2WI: Fat-suppressed T2WI
GPMN: Growth pattern of mural nodules
ICC: Interobserver correlation coefficient
NPV: Negative predictive value
OC: Ovarian cancer
O-RADS: Ovarian-adnexal reporting and data system
PCC: Pearson correlation coefficient
PPV: Positive predictive value
SE: Sensitivity
SP: Specificity
SVM: Support vector machine
T1WI: T1-weighted imaging
T2WI: T2-weighted imaging
