# Supplementary material for: The value of MRI in differentiating ovarian clear cell carcinoma from other adnexal masses with O-RADS MRI scores of 4–5
Source: Insights Imaging. 2025 Jan 29;16:22. doi: 10.1186/s13244-024-01860-z (PMC11780052; doi:10.1186/s13244-024-01860-z)
Supplement: Supplementary file 1 — ELECTRONIC SUPPLEMENTARY MATERIAL [file 13244_2024_1860_MOESM1_ESM.pdf]

The Value of MRI in Differentiating Ovarian Clear Cell Carcinoma from Other Adnexal Masses with O-RADS MRI Scores of 4-5

ELECTRONIC SUPPLEMENTARY MATERIAL

Table S1 Details of parameters for 1.5 Tesla MRI imaging protocols.

	Sagittal T2WI	Axial T1WI	FS-T2WI	CE-TIWI	DWI
TR(ms)	3100	500	3000	3.8	4260
TE(ms)	88.7	14.3	60	1.8	74.5
FA°	160	160	160	15	90
Band width (Hz/pixel)	35.71	31.25	50	62.5	250
Slice thickness (mm)	5	5	5	4	5
Echo spacing (ms)	89	14	60	2	75
Freq.FOV (mm)	28	40	40	40	40
Phase.FOV (mm)	1	1	1	0.8	1

Table S2 Details of parameters for 3.0 Tesla MRI imaging protocols.

	Sagittal T2WI	Axial T1WI	FS-T2WI	CE-TIWI	DWI
TR(ms)	4480	3.97	3740	5.92	7400
TE(ms)	104	1.29	92	2.85	69
FA°	120	9	120	9	
Band width (Hz/pixel)	710	1040	303	630	2540
Slice thickness (mm)	4	3	5	3	5
Echo spacing (ms)	7.44	8.4	7.9	15.4	0.49
FOV read (mm)	280	380	280	360	380
FOV phase(mm)	100%	81.3%	125%	81.3%	78%

Table S3 The interobserver agreement of the qualitative and quantitative MR features.

Qualitative feature	Kappa value	95% CI
O-RADS score	0.95	0.91-0.99
Shape	0.94	0.90-0.99
Parity	0.96	0.92-0.99
Unilocular	0.94	0.88-0.99
Component	0.94	0.89-0.98
Shape of solid	0.92	0.86-0.97
Growth pattern of mural nodules	0.85	0.78-0.93
High signal on T1WI	0.95	0.90-0.99
Quantitative feature	ICC value	95% CI
Minimum ADC value	1.00	1.00-1.00
Mean ADC value	1.00	1.00-1.00
Overall size(mm)	1.00	1.00-1.00
Solid portion size(mm)	1.00	1.00-1.00
Number of nodule	0.99	0.99-1.00
HWR	0.99	0.98-1.00
SSIR	0.97	0.97-0.98
FSIR	0.99	0.98-0.99
USIR	0.99	0.99-0.99

Note: The interobserver agreement was defined as slight agreement (0.00–0.40), moderate agreement (0.41–0.60), substantial agreement (0.61–0.80), and almost perfect agreement (0.81–1.00). O-RADS MRI score : the Ovarian-Adnexal Reporting and Data System MRI score; ADC: apparent diffusion coefficient 10^{-3} mm^2. HWR: meant the ratio of height to width of solid components; SSIR: solid signal intensity ratio; FSIR: fluid signal intensity ratio; USIR: uterine signal intensity ratio.

Table S4 Distribution of clinical and MRI characteristic parameters in training and testing sets

	Training sets (n =135)			Testing sets (n =86)			P-value
	Non-CCC (n =117)	CCC (n =18)	P-value	Non-CCC (n =74)	CCC (n =12)	P-value	
Age	56 (69)	55.5 (44)	0.74	56.59 (1.39)	50.92 (2.75)	0.11	0.51
Postmenopause	61 (52.1)	8 (44.4)	0.54	44 (59.5)	5 (41.7)	0.25	0.39
Endometriosis	5 (4.3)	7 (38.9)	0.001	6 (8.1)	7 (58.3)	0.00	0.15
CA125	248 (36235)	52.05 (601.76)	0.005	428.5 (9344.22)	40.35 (10193.3)	0.73	0.35
HE4	148 (3066.17)	59.25 (4177.8)	0.84	198.5 (637.8)	50.8 (280.7)	0.00	0.50
CEA	1.5 (318.7)	1.31 (5.19)	0.007	1.65 (35.1)	1.32 (3.07)	0.06	0.77
CA199	12.10 (9998.81)	15.95 (522.6)	0.18	13.22 (9998)	10.16 (883.66)	0.19	0.58
ROMA	0.65 (0.99)	0.25 (0.95)	0.02	0.85 (1)	0.09 (0.97)	0.00	0.36
Regular shape	44 (37.6)	10 (55.6)	0.15	28 (37.8)	10 (83.3)	0.003	0.54
Nultipara	72 (61.5)	15 (83.3)	0.7	39 (52.7)	12 (1)	0.002	0.44
Unilocular	27 (23.1)	13 (72.2)	0.00	17 (23)	10 (83.3)	0.00	0.78
Component			0.08			0.26	0.09
Cystic	24 (20.5)	6 (33.3)		9 (12.2)	3 (25)		
Solid	52 (44.4)	3 (16.7)		28 (37.8)	2 (16.7)		
Mix	41 (35)	9 (50)		37 (50)	7 (58.3)		
Shape of solid			0.14			0.31	0.37
Noudel	70 (59.8)	15 (83.3)		4 (64.9)	10 (83.3)		
Mass	26 (22.2)	1 (5.6)		11 (14.9)	0		
Mix	21 (17.9)	2 (11.1)		15 (20.3)	2 (16.7)		
Eccentric mural nodules	13 (72.2)	5 (27.8)	0.004	17 (23)	7 (58.3)	0.01	0.052
High signal on T1WI	32 (27.4)	14 (77.8)	0.00	10 (13.5)	9 (75)	0.00	0.06
Minimum ADC value	0.88 (1.06)	1.02 (0.91)	0.03	0.85 (0.91)		0.00	0.18
Mean ADC value	0.92 (1.04)	1.05 (0.92)	0.02	0.90 (0.85)		0.002	0.25
Overall size(mm)	700 (2900)	949 (1348)	0.29	700 (1870)	852.5 (2270)	0.06	0.49
Solid portion size(mm)	31 (848)	27.92(79.4)	0.36	30 (81.36)	26.21 (78.76)	0.98	0.62
Number of nodule	4 (19)	2 (9)	0.02	4 (14)	1.5 (11)	0.32	0.78
HWR	1.25 (1.9)	1.12 (1.46)	0.82	1.23 (2.24)	1.20 (1.91)	0.93	0.86
SSIR	0.48 (1.00)	0.44 (0.88)	0.57	0.49 (0.80)	0.46 (0.53)	0.66	0.27
FSIR	0.64 (5.38)	0.65 (2.5)	0.45	0.66 (2.84)	1.03 (1.13)	0.05	0.58
USIR	0.98 (1.84)	0.71 (1.67)	0.08	0.94 (1.51)	0.98 (0.66)	0.68	0.66

Note: continuous variables showed as medians (ranges), categorical variables showed as frequency (percentage); ROMA: meant the risk of ovarian malignancy algorithm; ADC: apparent diffusion coefficient 10^{-3} mm^2. HWR: meant the ratio of height to width of solid components; SSIR: meant solid signal intensity ratio; FSIR: meant fluid signal intensity ratio; USIR: meant uterine signal intensity ratio.
